# Fabrication of Porous Materials from Natural/Synthetic Biopolymers and Their Composites

**DOI:** 10.3390/ma9120991

**Published:** 2016-12-07

**Authors:** Udeni Gunathilake T.M. Sampath, Yern Chee Ching, Cheng Hock Chuah, Johari J. Sabariah, Pai-Chen Lin

**Affiliations:** 1 Department of Mechanical Engineering, Faculty of Engineering, University of Malaya, 50603 Kuala Lumpur, Malaysia; sampath@gmail.com; 2 Department of Chemistry, Faculty of Science, University of Malaya, 50603 Kuala Lumpur, Malaysia; chchuah@um.edu.my (C.H.C.); sabariah@gmail.my (J.J.S.); 3 Department of Mechanical Engineering, National Chung Cheng University, 621 Chiayi Country, Taiwan; impcl@ccu.edu.tw

**Keywords:** natural biopolymers, synthetic biopolymers, fabrication, biocomposites, porosity, sustainable

## Abstract

Biopolymers and their applications have been widely studied in recent years. Replacing the oil based polymer materials with biopolymers in a sustainable manner might give not only a competitive advantage but, in addition, they possess unique properties which cannot be emulated by conventional polymers. This review covers the fabrication of porous materials from natural biopolymers (cellulose, chitosan, collagen), synthetic biopolymers (poly(lactic acid), poly(lactic-*co*-glycolic acid)) and their composite materials. Properties of biopolymers strongly depend on the polymer structure and are of great importance when fabricating the polymer into intended applications. Biopolymers find a large spectrum of application in the medical field. Other fields such as packaging, technical, environmental, agricultural and food are also gaining importance. The introduction of porosity into a biomaterial broadens the scope of applications. There are many techniques used to fabricate porous polymers. Fabrication methods, including the basic and conventional techniques to the more recent ones, are reviewed. Advantages and limitations of each method are discussed in detail. Special emphasis is placed on the pore characteristics of biomaterials used for various applications. This review can aid in furthering our understanding of the fabrication methods and about controlling the porosity and microarchitecture of porous biopolymer materials.

## 1. Introduction

Biopolymers and their derivatives are abundant, varied, and important for living beings, they exhibit special properties and have greater importance for miscellaneous applications. These properties and the possibility of formation of these substances using renewable resources make biopolymers a popular initiative in industrial applications. In recent years, there has been an increasing interest in the use of biodegradable materials for packaging, medicine, agriculture, and other areas [1,2].

Biopolymers consist of monomeric units covalently bonded to form macromolecules. There are primarily two classes of biopolymers, namely, natural biopolymers and synthetic biopolymers. Natural biopolymers are obtained from living organisms and the synthetic biopolymers represent the macromolecules synthesized with biomolecules. Natural biopolymers are further divided into polysaccharides, proteins, polynucleotides, polyisoprenes, and polyesters. Synthetic biopolymers can be classified according to the way of preparation such as, biopolymers synthesized by addition and condensation polymerization reaction are listed separately [3]. Biocomposite materials are materials made from two or more constituent biomaterials that result in significant properties than those of the characteristics of individual components. Biodegradability and other properties of biopolymers strongly depend on the polymer structure. The properties of a polymer can be categorized into three broad classes: (1) intrinsic properties, which are inherent to the polymer itself; (2) processing properties, which refer to the behavior of material during forming; and (3) product properties in principle determined by combinations of intrinsic and processing properties. The practitioner needs more detailed information about processing properties such as viscosity, melt strength, melt flow index at the various stages of production [4,5].

Many of the applications of biopolymers can be found in the medical field, such as drug delivery systems, surgical implant devices, wound closure and healing products due to possessing of certain properties like, biocompatibility, biodegradation to non-toxic end products, high bio-activity, low antigenicity, ability to support cell growth and proliferation with appropriate mechanical properties, processability to complex shapes with appropriate porosity, as well as maintaining mechanical strength. Due to the film forming and barrier properties, biopolymers are widely used for applications including food containers, soil retention sheeting, agriculture film, waste bags and use as packaging material in general. They are also popular in areas such as automotive development, hazardous waste removal, paper industries and development of new building materials [6].

The introduction of porosity into a biomaterial broadens the scope of applications. Porous materials made from biopolymers having properties such as biocompatibility and biodegradability are of special interest for medical, cosmetic, pharmaceutical, and other applications. Three-dimensional scaffolds for tissue engineering, “green” packaging, delivery matrices and eco-friendly insulating materials are only a few examples of these applications [7].

Total porosity is one of the important structural parameters of a matrix to be used as a porous biomaterial. Total porosity is defined as the ratio of the total pore volume, to the overall (or bulk) volume. However, for certain applications, total porosity alone does not have a direct impact on its features. Pore size and pore interconnectivity are more important. Usually, high total porosity is accomplished by poor mechanical properties. Inside a porous biopolymer, there may be closed (isolated) pores and open (connected) pores. Pore interconnectivity is important for the accessiblity of gas, liquid, and particulate suspensions. Pore interconnectivity is defined as the ratio of the pore volume accessible from the matrix surrounding by a sphere of known diameter, to the total pore volume.

Apart from the total porosity and the pore interconnectivity, pore size and pore size distribution are also important for most kinds of applications. Pore size <10 µm, defined as micropores and pore size >50 µm considered as macropores.

**Figure 1:**
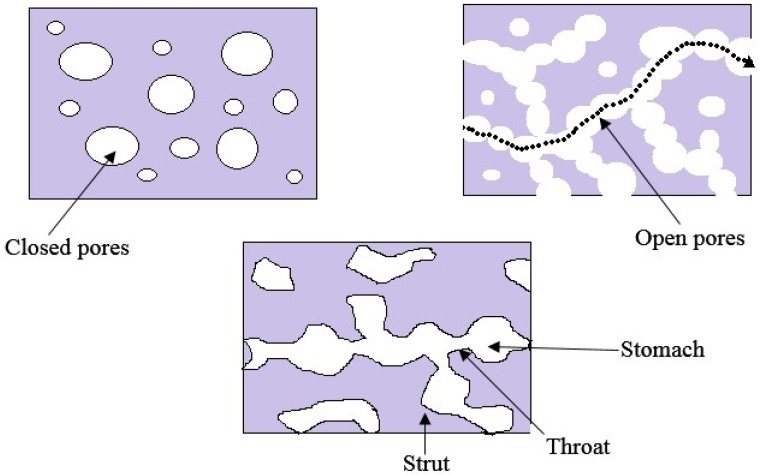
Porous features of porous biomaterial.

Figure 1 shows the porous features of a porous biomaterial. Micropores usually found in the struts of the porous biomaterials and the connected macropores are often irregularly distributed along the pathway. Therefore, there are two distinct apertures, namely throat and stomach. Pore morphology has a greater impact on tissue engineering applications as cell/tissue ingrowth behavior depend on porous structure. If the throat size is very small, cells and/or tissues are not able to penetrate or grow into the pores. Various methods have been developed for the fabrication of biomaterials. However, some processing methods do not guarantee high pore interconnectivity and large throat, even though a high total porosity is ensured [8].

Porous scaffolds can be manufactured using biopolymers with specific surface-area-to-volume ratio, crystallinity, pore size, and porosity. The three-dimensional porous scaffolds with enhanced porosities having a homogeneous interconnected pore network are required for tissue engineering applications. Ideal pore sizes vary for different cells and tissues. Porous controlled-release systems should contain pores that are sufficiently large to enable diffusion of the drug [9].

There are many techniques used to manufacture porous polymers. Those can be divided into two categories, designed manufacturing techniques and non-designed manufacturing techniques. Non-designed manufacturing techniques include freeze-drying or emulsion freezing, melt molding, phase separation, solvent casting or particulate leaching, gas foaming or high-pressure processing, electrospinning and combination of these techniques. Designed manufacturing technique includes 3D printing, rapid prototyping of solid free-form technologies. A modern method for creating porous structures using biodegradable fibers by electrospinning is the latest development in this field [9].

This paper aims to present a review regarding different fabrication methods used for the porous fabrication of biopolymers such as chitosan, cellulose, collagen, PLA, poly(lactic-*co*-glycolic acid) and their composite materials. Fabrication methods, including the basic and conventional techniques to the more recent ones, are tabulated. Despite significant development in fabrication methods, no single technique exceeds all the others, so this review will summarize the advantages and limitations of each method in detail.

## 2. Fabrication of Natural Biopolymers

### 2.1. Chitosan

**Figure 2:**
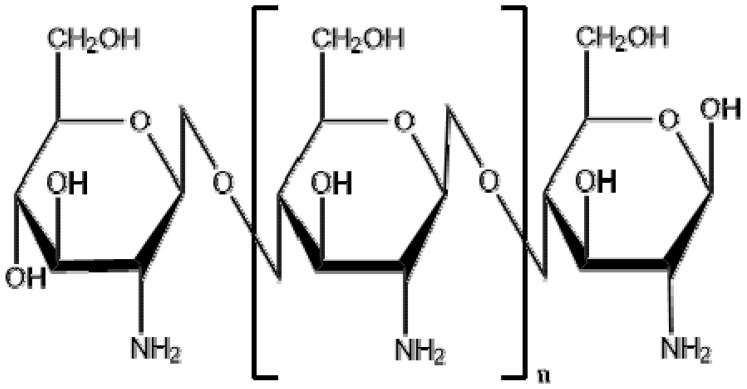
Chemical structure of chitosan [poly-(β-1/4)-2-amino-2-deoxy-d-glucopyranose].

Chitosan is produced by deacetylation of chitin, which is present in the exoskeleton of crustaceans (such as crabs and shrimps) and the bone plates of cuttlefish and squids. Chitin is recognized as second most abundant biopolymer in nature. Also, it is a major constituent of the cell wall of fungi. Due to the biological and mechanical properties of chitosan, it has been used to produce powder, hydrogels, membranes, fibers, porous scaffolds and beads that have been investigated with various biological and medical applications. High adaptability of chitosan for a vast range of applications is due to a high degree of chemical reactive amino groups present in d-glucosamine residues. When compared with the higher deacetylate chitosan scaffolds, lower deacetylate chitosan scaffolds possess smaller pore sizes, higher mechanical strength, moderate swelling properties and greater cellular activities [10]. The structure of chitosan is shown in Figure 2.

Chitosan porous membranes can be produced to be used as scaffolds by thermally induced phase separation method, in which temperature is decreased to freezing conditions, to induce the phase separation of the polymer. The pore size of the scaffold varied according to the temperature and water content. Low temperature and high water content will result smaller pore sizes. Hydrated porous chitosan membranes have a high surface area and volume when compared with the nonporous chitosan membranes, but their elasticity and resistant to the fraction is smaller than the nonporous membranes. Resistant and elasticity can be improved by the addition of glutaraldehyde, polyethylenglycol, heparin, or collagen, but this can make chitosan insoluble in acid solutions and to form closed pore structures [11,12].

Nwe, Furuike and Tamura [10] reported that porosity of the chitosan scaffolds produced by fungal mycelia was greater than that of the chitosan obtained from crab shells and squid bone plates. Chitosan from shrimp shells possessed the lowest porosity. During the scaffold construction process, when chitosan dissolved with acetic acid the molecular alignment disappeared and with freeze-drying step the formation of hydrogen bonds between polymer chains caused interconnecting of a network of pores. It was also observed that polygonal pores resulted when low molecular weight chitosan is used and elongated pores ensued from chitosan with high molecular weight. Porosity, pore size, and distribution pattern of pores highly affected the mechanical properties, water absorption, and water vapor permeability of the scaffold.

3D micro-porous chitosan scaffolds were produced by dissolving chitosan in acetic acid, followed by stirring and finally adding the solution into a NaOH solution. Here the foaming is achieved by mechanical stirring without adding a chemical foaming agent. It is mentioned that pore diameter of chitosan scaffold decreases with increasing the stirring rate of the sample due to the high shear force of the homogenizer’s cutting head. Also, the pore diameter decreased with decreasing the concentration of chitosan in the solution. Concentrations of chitosan solutions less than 1%, lowered the sample’s viscosity for foaming and on the other hand concentrations greater than 3%, caused sample’s viscosity too high and stick to the homogenizer’s cutting heads which resulted in difficulties in mixing [13].

Porous chitosan matrices can be used for the removal of heavy metals from contaminated water. Chitosan and chitosan modified with glutaraldehyde microparticles were prepared for Pb(II) biosorption and compared the properties such as morphology of particles, solubility and pore characteristics. It is reported that glutaraldehyde crosslinked chitosan produces more interconnected pores than that of chitosan microparticles. Porosity increased with the proportion of glutaraldehyde used for the crosslinking. The proportion of the crosslinking agent was found to have no effect on particle size. However, the surface morphology was found to be changed with the crosslinking agent [14].

Formation of a nonporous skin layer is a barrier to the cell growth in tissue engineering scaffolds. Chitosan hydrogels were fabricated with dense gas CO_2_ and porous structure on top, bottom surface and in cross sections were obtained without formation of a nonporous skin layer. Due to the high porosity, the crosslinked chitosan hydrogel fabricated under dense gas conditions showed higher equilibrium swelling ratio than the samples fabricated under atmospheric conditions. Hydrogels exhibited equilibrium swelling ratios of 17.2 ± 0.8 and 10.3 ± 0.4 at the dense gas condition and atmospheric condition, respectively [15].

Low viscosity and high diffusivity of supercritical CO_2_ offers fabrication of highly porous tissue engineering scaffolds. Chitosan hydrogel was fabricated using supercritical CO_2_ for the implantation of osteoblast cells. Hydrogel fabricated under 250 bar, 45 °C, 2 h at 5 g/min CO_2_ flow rate yielded 87.03% porosity which was similar to lyophilization (88.68%) operated at 55 °C for 48 h. Even though the porosity is similar for both conditions, the fabrication using supercritical CO_2_ found to be more time and energy efficient [16].

Macroporous chitosan scaffolds were developed to load either with bone morphogenetic proteins (BMP-2) or insulin-like growth factor (IGF-1) to study the bone healing property in vivo. Porosity was developed by mechanical stirring the chitosan solution using homogenizer and transferring the bubbled solution into sodium hydroxide solution to conduct liquid hardening process. Chitosan scaffold with pore sizes from 70 to 900 µm was obtained using liquid hardening method. The absorption efficiency of IGF-1 and BMP-2 was found to be 90% ± 2% and 87% ± 2%, respectively. In vivo studies revealed that chitosan scaffolds loaded with IGF-1 showed significant osteoblastic differentiation than BMP-2-loaded chitosan scaffolds [17].

### 2.2. Cellulose

**Figure 3:**
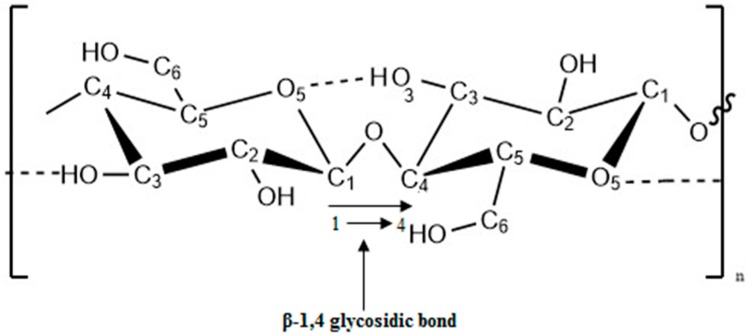
β-1,4 glycosidic bond of a cellulose unit.

Cellulose is the most abundant organic polymer on earth. It is an important structural component of the primary cell wall of plant cells and tissues [18]. The building block of the cellulose polymer is monosaccharide glucose molecules. Polymer consists of repeated glucose units attached together by β-1,4 glycosidic linkages as shown in Figure 3. β-1,4 glycosidic bond is formed by covalent bonding of oxygen to the C1 of one glucose ring and the C4 of the connecting ring. Three hydroxyl groups containing in the repeating unit and their ability to make hydrogen bonds between cellulose chains responsible for the physical properties of cellulose [19,20,21].

Cellulose can be synthesized by fungi and some species of bacteria (*Acetobacter xylinum*). Bacterial cellulose is similar to plant cellulose in chemical structure, but the lack of contaminant molecules (lignin and hemicelluloses). Hence, it does not require intensive purification methods. Due to the significance of mechanical strength and biocompatibility, cellulose is widely used in tissue engineering applications [22].

Cellulose is used as a raw material in industries such as veterinary, foods, fibers, textile, wood, paper, cosmetic and pharmaceuticals. Derivatives of cellulose also play a major role in the applications of fibers, textiles, coatings, thermoplastic films, pharmaceutical technologies and as food additives [21]. Development of highly porous structure in cellulose is important because of their potential uses in biomedical applications, filtration, controlled flow of fluids, aircraft, automotive, building and packaging industries [23].

Foams are closed pore structures with the presence of cavities that are not interconnected. These foam structures used to enhance lightness, impact strength, softness, and thermal insulating properties in automotive, aircraft, building and packaging industries. The porous structure can be produced by introducing a blowing agent into the polymer solution. Supercritical CO_2_ is widely used in medical applications as CO_2_ can be completely removed from the product. In supercritical CO_2_ process, rapid depressurization rates cause homogeneous pore distributions with closed pores. Decreasing the depressurization rates will produce wide and large pore size distributions, and more interconnected pores [23,24].

Direct addition of antimicrobial agents to initial food formulations may decrease its concentrations on the food surface due to its diffusion into inner parts of the food. Also, it may cause neutralization of the agent due to interaction with the constituents of food. Controlled release of antimicrobial agent can be achieved by loading it into the food packaging material. Baldino, et al. [25] produced cellulose acetate antimicrobial membrane using supercritical CO_2_ as pore forming agent. Cellulose acetate dissolved with the antimicrobial agent put into the membrane-preparing vessel and filled with supercritical CO_2_. The vessel was then flushed with CO_2_ and depressurized for about 30 min. They observed that the mean pore size decreased with increasing the operative pressure. It was also observed that the pore size decreased with decreasing the operative temperature.

Freeze-drying process and introducing of pore forming chemicals were successfully tested for the creation of porous structure in cellulose materials for building insulation applications. The introduction of various substances, which producing CO_2_ such as brewer’s yeast, baking powder and NaHCO_3_ have been tested for foam formation in cellulose matrices. Porous structures in cellulose insulating materials were also obtained by the sublimation of water during the freeze-drying process [19].

Three-dimensional macroporous scaffold from bacterial cellulose was developed for culture of breast cancer cells and patterned pores were created by using an infrared laser. Different pore sizes were able to fabricate by adjusting the distance between specimens and laser focus. In fact, that the cancer cells were larger than the tissue cells, satisfactory biocompatibility was obtained with the macroporous scaffold produced by bacterial cellulose [26].

### 2.3. Collagen

Collagen is the most abundant structural protein in the vertebrate body. It is a major component of connective tissues, skin, bone, cartilage, and tendons. Moreover, collagen is the most abundant protein type of the extracellular matrix of connecting tissues, which provides structural integrity and conferred the mechanical and biochemical properties. At present, 28 types of collagen have been identified, and among these, the dominant collagen present in extracellular matrix, in tissues such as skin, tendon and bone is type I collagen. Type II collagen found in cartilage and type III occurs in adult skin [27].

**Figure 4:**
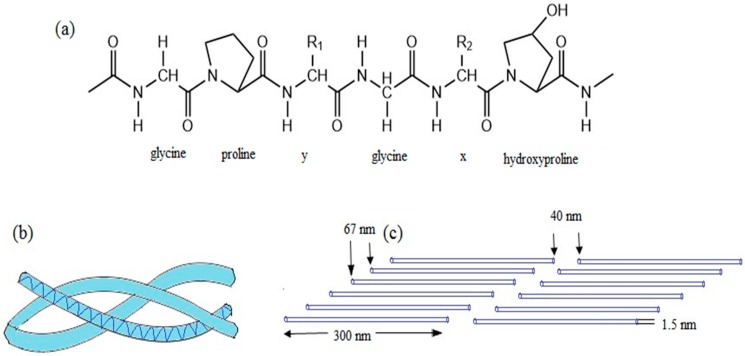
Chemical structure of collagen type I (**a**) Primary amino acid sequence; (**b**) secondary left handed helix and tertiary right handed triple-helix; (**c**) staggered quaternary structure.

Collagen protein has a complex hierarchy of structural order in primary, secondary, tertiary and quaternary structures. In primary structure, every third amino acid is a glycine, with strict repeating as shown in Figure 4a. About 35% of the non-glycine positions in the repeating unit are consist of proline, can be mostly found in x-position and 4-hydroxyproline, predominant in y-position. In secondary structure, glycine and hydroxyproline units lead to form a helical macromolecule. In tertiary structure, three helical units twist to form a right-handed triple-helical collagen molecule as shown in Figure 4b. In quaternary structure, triple-helical collagen molecules stagger into fibrils, which then arranged into fibers or even larger fiber bundles as illustrated in Figure 4c [28].

Collagen is important as a biomaterial for various applications due to its specific properties such as abundance, biocompatibility, high porosity, easy processing, a facility for combination with other materials, low antigenicity, hydrophilicity and absorbability in the body. Collagen can be processed into various forms such as sheets, sponges, tubes, fleeces, powders, injectable solutions and dispersions, which then can be used as systems for drug delivery, scaffolding materials which promoting cell migration, wound healing, and tissue regeneration. Various methods have been developed for the modification of processing characteristics of collagen, including porosity development to suit with a wide range of applications [29].

Oh, et al. [30] developed porous collagen scaffold with using ice particulates as templates. Templates were prepared by dispersing the water droplets on a cooled copper plate wrapped with perfluoroalkoxy film. Ice templates were cooled to −5 °C and introduced the collagen solution followed by gradual freezing to −5 °C. Finally, ice crystals were removed by freeze-drying and micropatterned pores were obtained. Two types of pores were obtained; one is the negative replica of ice templates and other is from ice crystals developed from freeze-drying. Pore sizes were able to control by freezing temperatures. Decreasing the freezing temperature produced scaffolds with smaller pores.

Collagen scaffold with controlled insulin release properties was developed with freeze-drying method for cartilage tissue engineering. Insulin was microencapsulated with poly(lactic-*co*-glycolic acid) beads and introduced to the collagen aqueous solution. Prepared ice particulates were added to the collagen bead mixture solution and freeze-dried to obtain the required pore size of the scaffold. Scaffold with interconnected pore structure was obtained with pore sizes equivalent to ice particulates. Drug release studies revealed that the scaffold exhibited a zero order release kinetics of insulin up to a period of 4 weeks [31].

Highly porous hydrogel with enhanced salt and pH resistance properties was prepared using the hydrolyzed collagen as the backbone of the hydrogel. Acrylic acid and 2-acrylamido-2-methylpropanesulfonic acid were polymerized and crosslinked to hydrolyze the collagen backbone. The porous structure was achieved by partially neutralizing the grafted polymer after gel formation. The pores formed in the gel due to the water evaporation as a result of neutralization heat [32].

## 3. Fabrication of Synthetic Biopolymers

### 3.1. Poly(lactic acid)

Among the biobased materials, poly(lactic acid) (PLA) is one of the most promising biomaterials and has remarkable properties, which make it suitable for different applications. It is cheaper and commercially available with wide range of grades. Since the basic monomer unit (lactic acid) synthesized by the fermentation of renewable resources (carbohydrates), PLA complies the concept of sustainable development and is classified as an eco-friendly material [33].

**Figure 5:**
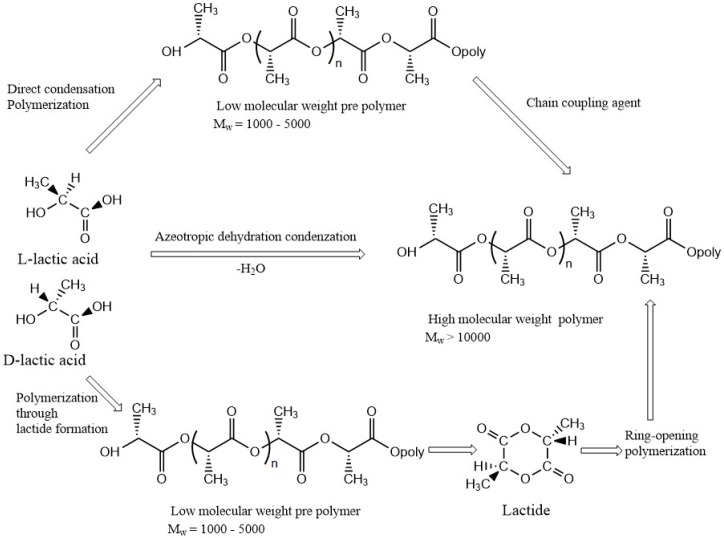
Three main routes for obtaining high molecular weight poly(lactic acid) (PLA).

Figure 5 shows the three main routes for the synthesis of PLA. In the first step, lactic acid is condensation-polymerized to form low molecular weight prepolymer and employed with chain coupling agent to increase chain length and to form high molecular weight PLA. The second step involves azeotropic dehydrative condensation, a one-step process to form high molecular weight PLA from the monomer unit. The third and the main process is a ring-opening process of lactide into high molecular weight PLA which is patented by Cargill (US) in 1992 [34].

PLA has been widely used for tissue engineering applications due to its biocompatibility, biodegradability and ease of fabrication into porous structures. Conde, et al. [35] developed a poly(l-lactic acid) scaffold using solvent-casting/particulate leaching to investigate the influence of pore size on the proliferation and differentiation of dental pulp stem cells. The scaffolds were prepared in pulp chambers of 1 mm thick tooth slices and porosity was developed using salt crystals of two sizes (150–250 µm or 251–450 µm) as porogen. It was observed that scaffolds with different pore sizes allowed the proliferation and differentiation of dental pulp stem cells into odontoblast-like cells.

PLA tubes were fabricated by atomizing the polymer solution over a rotating shaft. Polymer solution was prepared using a mixture of ethanol and dichloromethane. Due to the low boiling point of dichloromethane, it evaporated before ethanol. Also, dichloromethane is a good solvent for PLA than ethanol. Then ethanol would become more concentrated and reached to the critical concentration stage where PLA became to precipitation. At last, ethanol evaporation took place and created PLA tubes with internal pore structure with a rough surface [36].

Room temperature ionic liquid based on 1-butyl-3-methylimidazolium bearing hydrophilic anion Cl was used for the preparation of three-dimensional (3D) porous PLA scaffold for tissue regeneration. Hydrophobic PLA and relatively hydrophilic room temperature ionic liquid containing anion Cl was dissolved in dichloromethane and formed a homogeneous mixture. It later formed a continuous bicomponent network by the phase separation process. After the ethanol washing step, complete removal of room temperature ionic liquid created the pore structure in the biopolymer scaffold. Open porous PLA network with pore sizes greater than 100 µm and porosities of about 86%–94% was obtained by using this method [37].

Gelatin particles were used as a porogen for the fabrication of poly(l-lactic acid) (PLLA) scaffolds for chondrocyte regeneration. Gelatin particles (280–450 µm) sieved from raw gelatin was boned by incubation in saturated water vapor and immerged into PLLA/1,4-dioxane solution. After freeze-drying, gelatin immerged PLLA/1,4-dioxane solution was treated with water to remove the gelatin particles to obtain the porous scaffold. Scaffolds with interconnected pore structure and pre-designed pore sizes (280–450 µm) with the porosity >94% were developed by using this method [38].

Solid state foaming of biomaterials can be achieved by using gasses such as N_2_ and CO_2_. However, the main drawback of this method is, it formed mostly closed pores, which are not suitable for applications such as tissue engineering. A method was developed to fabricate PLA using solid-state foaming and ultrasound for tissue engineering applications. CO_2_ blowing was carried out at room temperature with gas pressures at 3–5 MPa. Ultrasound was applied to break the pore walls of the solid state foams (closed pores). It was observed that ultrasound can successfully apply to improve the interconnectivity of pores of PLA created by CO_2_ blowing method [39].

The porous scaffold of PLA was developed by applying solid state extrusion combined with porogen (NaCl) leaching method. Poor mechanical properties exist in porogen leaching method to porous scaffold was overcome by utilizing solid state extrusion process. The introduction of biocompatible poly(ethylene glycol) (PEG) as a plasticizer caused the development of PLA ductile and enabled the formation of interconnected pores. Highly interconnected porous architecture with high connectivity exceeding 97% and with enhanced porosity over 60% was obtained in PLA scaffold with the composition of NaCl higher than 75.00 wt % and PEG more than 1.25 wt % [40].

### 3.2. Poly(lactic-co-glycolic acid)

**Figure 6:**
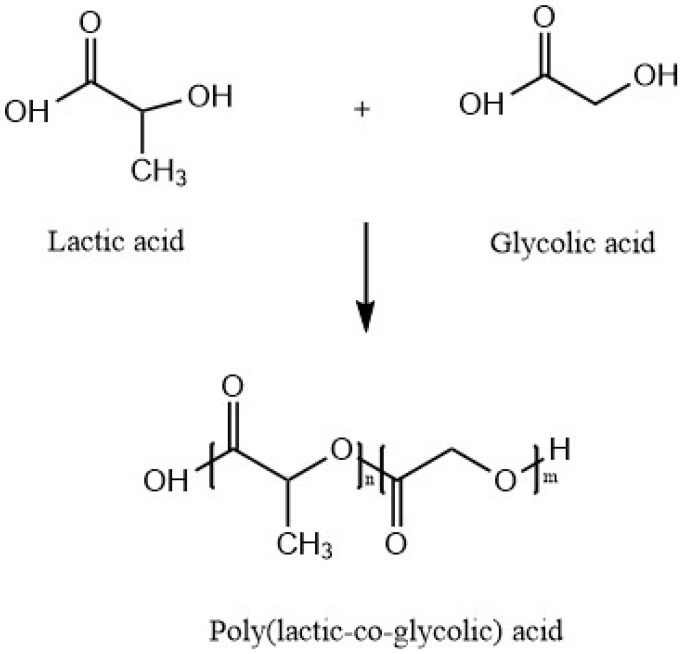
Poly(lactic-*co*-glycolic acid) (PLGA) and its constituent monomers, lactic and glycolic acid.

Poly(lactic-*co*-glycolic acid) (PLGA) is a copolymer synthesized by means of ring-opening *co*-polymerization of poly(lactic acid) (PLA) and poly(glycolic acid) (PGA). Since PLA contains an asymmetric α-carbon, it is typically described by two enantiomeric forms of poly(d-lactic acid) (PDLA) and poly(l-lactic acid) (PLLA). PLGA is known as poly(d,l-lactic-*co*-glycolic acid) when poly(d-lactic acid) (PDLA) and poly(l-lactic acid) (PLLA) are present in equal ratios. PLGA also can be prepared by different ratios of its monomeric units. Different types of PLGA can be obtained by the different ratios of monomer units. These are identified according to the ratio of two types of monomers. For instance, PLGA 75:25 refers to a copolymer consists of 75% lactic acid and 25% glycolic acid [41]. Figure 6 shows the structure of PLA and its constituent monomers, lactic and glycolic acid.

Unlike the two types of monomers, PLGA dissolved in a wide range of common solvents such as tetrahydrofuran, chlorinated solvents acetone or ethyl acetate. PLGA degrades by hydrolysis in aqueous environments and produces lactic acid and glycolic acid as byproducts. Degradation rates depend on the molecular weight of the polymer, the ratio of glycolic acid to lactic acid, stereochemistry (depends on the d and l-lactic acid monomers), and end group functionalization (polymers end capped with esters degrades slowly than the presence of free carboxylic acid groups at the end). Since the *T*_g_ is above 37 °C, PLGA shows a glassy behavior in nature. It is reported *T*_g_ decreases with decreasing the amount of lactic acid present in the copolymer [42].

Due to biocompatibility, tailored biodegradation, potential to modify surface properties and ease of fabrication into porous structures, PLGA is being considered and investigated for a wide range of biomedical applications [42]. They are mainly used in the pharmaceutical industry to develop drug delivery systems, as a sutures for wound closure and as scaffolding materials for tissue engineering [43].

Krebs, et al. [44] developed an injectable, PLGA scaffold with in situ pore formation via phase inversion. Porogen and a small amount of water were introduced into the PLGA polymer solution for the in situ pore formation, as with previous studies, a porous structure was not given alone with the polymer solution. When water-insoluble PLGA contact with the aqueous solution, it precipitated by phase inversion. Due to the porogen and the small amount of water, it created a microporous interconnected architecture on the surface and within the bulk.

PLGA foams were produced by the pressure quench method using supercritical CO_2_ as the blowing agent. Due to the prolonged exposure of the polymer to supercritical CO_2_, it decreased its glass transition temperature than the processing condition of the vessel and made a polymer/gas solution. After the rapid depressurization, the solubility of CO_2_ decreased with the polymer and caused bubble nucleation due to the supersaturation. This resulted in interconnected PLGA foams with relative pore densities ranging from 0.107 to 0.232 and porosities as high as 89% [45].

Thermally induced phase separation method was used to fabricate PLGA tubular foam scaffold for tissue engineering applications. The polymer solution was filtered using 0.45 µm nylon filter and cast to a petri dish. Then frozen in liquid nitrogen, and the solvent was sublimated in a vacuum. Porosity >93% with macropores of ~100 µm average diameter and interconnected micropores of 10–50 µm diameter were obtained with this method. Composition and porosity can be tightly controlled by thermally induced phase separation method. This ensures the optimization of scaffold for particular tissue engineering application [46].

Porous PLGA microparticles were prepared by water-in-oil-in-water (W_1_/O/W_2_) multi-emulsion method, to study the pH related drug release properties. Tiotropium (drug) dissolved in sodium tetraborate aqueous solution with a porogen and pH-sensitive drug release activator (3-diethylaminopropyl-conjugated hyaluronate) (W_1_ phase) and vigorously emulsified with PLGA dissolved in dichloromethane (O phase). The mixed solution was injected to polyvinyl alcohol and NaCl aqueous solution (W_2_ phase). After that, emulsification was carried out in homo-mixer at 4000 rpm for 5 min and hardened by mild stirring for 1 h at 50 °C. Microparticles were collected by centrifugation. The average pore diameters of the microparticles with 0, 20 and 50 mg of pH-sensitive drug release activator at pH 7.4 were 11, 12, and 27 nm, respectively. However, the average pore diameters of the microparticles with 0, 20 and 50 mg of pH-sensitive drug release activator at pH 6 were 13, 23, and 120 nm, respectively. These results indicated that the incorporation of pH-sensitive drug release activator increased the average pore diameter and surface area of microparticles in acidic medium [47].

**Figure 7:**
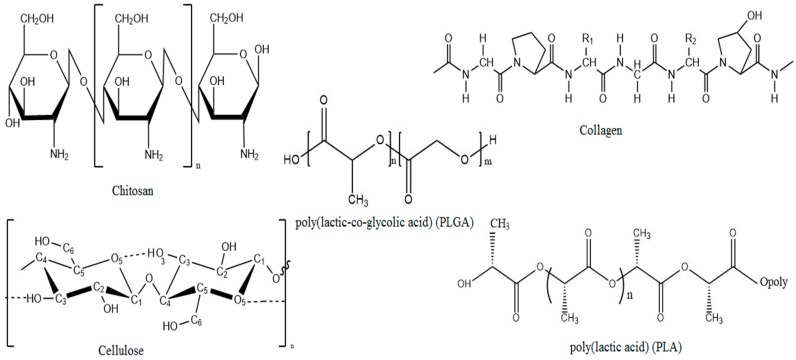
Chemical structures of biopolymers commonly used in the preparation of porous biomaterials.

Chemical structures of biopolymers commonly used in the preparation of porous materials are shown in Figure 7. The pore characteristics of biomaterials formed by natural biopolymers and fabricated with different methods are listed in Table 1.

## 4. Biocomposite Materials

Composite material is a material made from two or more constituent materials that result in significant properties than those of the characteristics of individual components. Composites can be produced with the view of tailoring physical, chemical, or mechanical properties, to fulfil the requirements of different applications such as automotive, packaging, aeronautic, naval, and so on [48,49].

In view of their potential for high performance, composite biomaterials have been studied and tested for various kind of applications as shown in Table 2. To be considered as biocomposite material, each constituent of the composite must be biocompatible. Extreme modifications of the properties of biomaterials can be improved by incorporation of nanosized filler. This properties improvement depends on both nanofiller geometry and on the surface (interface) area. There are three types of nanofillers namely, spherical, layered and acicular, based on their aspect ratio and geometry. A wide range of nanobiocomposites has been produced by introducing nanosized fillers and tested to overcome the conventional drawbacks of biopolymer [50].

To be successful for the applications, any biomaterial requires a wide range of study about its fabrication and properties. Fabrication of porous structures in biocomposites with various material combinations has become an increased research interest due to their wide range of applications in areas such as tissue engineering, nanocomposites, drug delivery systems, packaging and the automotive industry [51].

### 4.1. Fabrication of Porous Biocomposite Materials

#### 4.1.1. Chitosan/PLA Composite Materials

PLA is biocompatible and undergoes scission in the body to form lactic acid, which is a natural intermediate in the process of metabolic conversion of carbohydrates. These characteristics make this polymer compatible for use in biomedical applications. However, PLA has several drawbacks such as acidic degradation product, past biodegradation, and hydrophobicity. Chitosan is another biodegradable and biocompatible material, hydrophilic and alkaline in nature, which is widely used in biomedical applications. Acidic byproduct produced by PLA can be readily neutralized due to the alkaline nature of chitosan when using these two polymers as biocomposite material [52].

Li, Ding and Zhou [52] obtained the porosity of Chitosan/PLA scaffold by introducing NaCl to the mixture of chitosan and PLA. The mixture was then melted at 160 °C and molded, after that, NaCl grains were excluded by dipping the composite material in distilled water. Porosity was able to regulate by controlling the weight fraction of NaCl.

The blending of chitosan with PLA is difficult as chitosan decomposes before melting, due to high glass transition temperature of chitosan. Also, it is difficult to find a cosolvent to dissolve these two substances as chitosan dissolves in very few dilute acids and PLA only dissolves in few organic solvents. Therefore, methods were developed to grafting lactic acid onto the amino groups in chitosan by vacuum freeze-drying and vacuum reaction without a catalyst. It was observed that the porosity decreased with increasing lactic acid/chitosan feed ratio. Also, a higher porosity with large pores was formed with chitosan than with the PLA in the grafted copolymer [53].

By mixing of chitosan with PLA, formed a composite material with improved intensity and elasticity, to be used as nerve conduits. The chitosan and PLA solutions were mixed with best constituent ratios and sprayed over a rotating glass rod mandrel with compressed nitrogen gas. Infrared rays were used for dehydration and solidification. The detached coatings were sterilized and used as conduits. The conduits were biodegradable and provided many micropores to enhance the permeability. The pore size of chitosan/PLA conduits were less than 1 µm which allowed the permeability only for nutrient, other molecules and not for other cells [54].

Electrospinning is a method of production of polymer fibers in the diameter range from few nanometers to several micrometers. Also, electrospinning is used to form nanofibers with large surface-area-to-volume ratio and high porosity with small pore size. PLA/chitosan composite nanofibers were produced using coaxial electrospinning method and tested the potential of application as porous antibacterial material using antibacterial test. Results showed that antibacterial effect depended on the fabrication core feed rate [55].

#### 4.1.2. PLA/Cellulose Nanocomposites

Due to the biocompatibility, PLA is widely used in biomedical applications. The degradation product, lactic acid is also biocompatible due to the possibility of incorporation into the carbohydrate metabolism. However, due to the poor mechanical properties of PLA, various types of fillers such as carbon fibers, carbon nanotubes, hydroxyapatite and cellulose microcrystals have been introduced for the reinforcement of the polymer [56].

Cellulose nanocrystals, also known as nanowhiskers, have been introduced as a filler for the polymers due to exceptional mechanical properties, high aspect ratio, and large specific surface area. Cellulose nanowhisker can be obtained from plant cellulose as well as bacterial cellulose. They are exceptionally hydrophilic and shows high tensile strength properties [56].

Porous bacterial cellulose nanowhisker/PLA composites were fabricated using solvent casting and freeze-drying method to evaluate its possibility to use as tissue engineering scaffold and drug carriers. Bacterial cellulose nanowhisker suspension in water was introduced to PLA dissolved in 1,4-dioxane. After that, the mixture was frozen in liquid nitrogen and then freeze-dried to remove most of the 1,4-dioxane and water to obtain the porous composite. It was observed that the porosity increased from 79% to 92% by adding 5 wt % of bacterial cellulose nanowhisker to PLA [57].

Producing of polymer foams using supercritical CO_2_ was successfully tested for various kinds of applications such as large volume packaging applications and biomedical implants. Cell size and cell density of the foams of PLA produced using supercritical carbon dioxide were affected by the addition of cellulose nanofibers. Cellulose nanofibers were processed for surface modification, prior to mixing with the polymer in order to enhance the binding affinity. A mixture of PLA and cellulose nanofiber was prepared by hot pressing and foams were obtained by pressure quenching of supercritical CO_2_. Results showed that the foam cell size decreased and cell density increased with increasing the cellulose nanofiber concentration [57].

The role of cellulose nanofibers in the supercritical foaming process of PLA was investigated with different loadings of the nanofibers. Nanocomposite sheets were obtained using a casting–kneading–hot pressing procedure. Supercritical CO_2_ was used as a plasticizer and blowing agent. The presence of cellulose nanofibers speeded up the nucleating process, but lowered the cell growth rate and delayed the coalescence. Also, the addition of cellulose nanofibers reduced the cell size and increased the cell density of the foam [58].

PLA/cellulose nanocrystal nanocomposite fibers produced by electrospinning method was investigated for control release of nonionic compounds with varying the concentration of cellulose nanocrystals (0, 1, and 10 wt %). The incorporation of cellulose nanocrystals increased the crystallinity of PLA, accelerated the hydrolytic degradation and decreased the hydrophobicity of the polymer. At the same time, the mean pore size of the composite material was increased with increasing the cellulose nanocrystals. Due to the bigger pore size and high water uptake with 10% cellulose nanocrystals incorporated PLA, favored higher diffusion, and hence, increased the nonionic compound release percentage [59].

#### 4.1.3. Cellulose/Chitosan Composite Materials

Cellulose is polysaccharide made from glucose subunits and chitosan is made from amino polysaccharide subunits. The chemical structure of chitosan backbone is very similar to that of cellulose. Therefore, studies have been done to test the miscibility of chitosan with cellulose and introduction of amino groups to cellulose to improve the physical, chemical, mechanical and biological properties of developed composites [55].

Spray dried chitosan mixed with cellulose using *N*-methylmorpholine-*N*-oxide and deodorizing properties, metal ion sorption properties were investigated with varying concentrations of both polymers. A mixture of two polymers extruded from a syringe to form droplets and lyophilized until drying. Lyophilization process and freezing created open pores with a high degree of interconnectivity. Pore diameter decreased with increasing the chitosan concentration. This was due to the increase in interactions among chitosan, cellulose, and water, hence, decrease in ice crystal growth. Increasing the concentration of chitosan contributed to increased sorption of deodorizing agent and metal ions due to high interactions of these particles with chitosan than cellulose [60].

In contrast to the cellulose derived from plants, bacterial cellulose is free from contaminants such as natural fiber like lignin and hemicellulose. Low molecular weight chitosan was introduced to bacterial cellulose produced by *Acetobacto xylinum* and properties were modified with varying concentration of chitosan with different molecular weights. Chitosan was directly introduced to the culture medium and the product formed with bacterial cellulose was separated as sheets. It was observed that the pore size and surface area of the dried films were increased with the addition of low molecular weight chitosan. Also, the mechanical properties and water absorption capacity increased with the introduction of chitosan, but water vapor transmission rates, average crystallinity index and anti-microbial ability remained unchanged [61].

Chitosan/cellulose hydrogel beads were prepared using ionic liquid and investigated the dye adsorbent properties with varying dye concentrations, the number of beads and initial pH. The pore diameters of the beads were within the range of 10 to 20 nm. The maximum adsorption capacity of hydrogel beads was found to be 40 mg/g, for congo red dye removal from aqueous solutions, which was more efficient when compared with other economical methods [62].

Bacterial cellulose and chitosan composite material for potential biomedical application was produced by immersion of wet bacterial cellulose pellicle in chitosan solution followed by freeze-drying method. A very well interconnected porous network structure with larger aspect surface, which suitable for cell adhesion and proliferation, was obtained with using this method. Cell adhesion studies revealed that the biocompatibility increased with the addition of chitosan to the bacterial cellulose [63].

#### 4.1.4. Chitosan/PLGA Composite Materials

Due to the hydrophobic characteristics of PLGA, it is difficult to apply alone as a biopolymer in many applications like tissue engineering. To overcome these difficulties, composite materials have been made with PLGA by mixing with a hydrophilic biopolymer. Due to the presence of β-(1-4)-linked between d-glucosamine and *N*-acetyl-d-glucosamine and the ability of d-glucosamine to immobilization of ligands and glycoproteins through covalent bonding, chitosan has been widely used to mix with PLGA to make biocomposite materials, especially related to tissue engineering applications [64].

Kim, Yang, Chun, Chae, Jang and Shim [64] prepared a chitosan/poly(d,l-lactic-*co*-glycolic acid) composite fibrous scaffold by the coelectrospinning process and compared the properties with chitosan and PLGA scaffolds. Fibrous scaffold with high surface-to-volume ratio, high porosity, and variable pore size distribution was obtained by electrospinning method. The weak mechanical properties of chitosan were improved by electrospinning with PLGA; on the other hand, chitosan provided the hydrophilic property to PLGA. In the electrospinning process, the spinning parameters, solution viscosity, polymer concentration, applied voltage, and flow rate greatly influenced the porosity and pore size distribution of the composite material.

Chitosan/PLGA nanocomposite scaffold was produced via electrospinning and unidirectional freeze-drying techniques for tissue engineering applications. The porosity of composite material was found to be more than 96% and it decreased with increasing the chitosan concentration. In addition, the scaffold exhibited high surface area-to-volume ratio due to the incorporation of PLGA nanofiber [65].

Porous chitosan/poly(d,l-lactic-*co*-glycolic acid) nanocomposite scaffold was prepared using electrospinning and freeze-drying method and properties were studied with varying processing parameters, such as electrospinning time and the concentration of chitosan solution. The water absorption capacity and porosity of the nanocomposite scaffold decreased with an increase in the chitosan solution concentration and electrospinning time. In addition, compressive strength and the modulus of compressibility of the nanocomposite material improved due to the introduction of PLGA nanofibers [66].

Ajalloueian, et al. [67] developed novel nanofibers using a blend of chitosan and PLGA through emulsion electrospinning method for biomedical applications. Polyvinyl alcohol was introduced to the electrospinning solution as an emulsifier. After that, the polyvinyl solution was removed by extraction with ethanol solution. Porosity and hydrophilicity of the composite membrane increased due to the removal of polyvinyl alcohol from the mats. The improved hydrophilicity was expected to lead to higher cell affinity of the composite scaffold.

#### 4.1.5. Chitosan/Collagen Composite Material

Collagen is a one of promising biomaterial used in tissue engineering applications due to its superior biocompatibility and biodegradability. The main difficulty with collagen as a tissue engineering scaffold is rapid degradation when exposure to body fluids and cell culture media. Chitosan has been widely used in biomedical applications due to many advantageous such as antibacterial activity, wound healing property, and accelerating tissue regeneration. In addition, chitosan can function as a bridge to improve the mechanical strength of collagen scaffolds due to the presence of a large number of amino groups attached to its backbone [68].

Chitosan/collagen scaffolds were fabricated using freezing and lyophilizing methods with varying concentration of both biomaterials. Results showed that the pore size of the composite scaffold was comparatively smaller than chitosan scaffolds. Porosity decreased with increasing the concentration of chitosan but remained unchanged with the concentration of collagen. Moreover, the addition of collagen decreased the mean pore size of the scaffold, which is expected to improve the binding capacity of fibroblasts [68].

Chitosan/collagen scaffolds were formed using glutaraldehyde (GA) as a crosslinking agent and freeze-drying method for skin tissue engineering applications. It is reported that the mean pore size of crosslinked scaffold is higher than the pore size of the uncrosslinked scaffold. Rehydration and relyophilization process accompanied with GA cross-linking treatment further increased the pore sizes due to the fusion of smaller pores and by inducing the combination of collagen fibers [69].

Porous collagen membranes were placed in a chitosan solution and crosslinked with glutaraldehyde vapor to obtain chitosan-coated collagen membrane. Chitosan solution was added on collagen membrane without applying any pressure to allow the chitosan to penetrate into the pores of the collagen membranes. Results showed that the pore size and the porosity decreased in coated collagen scaffolds when comparing with the non-coated collagen scaffolds [70].

Most common method to create chitosan/collagen composite scaffold is freeze-drying process. Chitosan/collagen matrices often need an additional crosslinking step to increase the mechanical strength, stability and to prevent from degradation. Glutaraldehyde is the most commonly used crosslinking agent for both chitosan and collagen due to its high effectiveness. In most cases, cells cannot be incorporated into the matrix during the fabrication stage due to the high cytotoxicity of glutaraldehyde, and therefore it must be implanted into the exterior of scaffolds post-fabrication. Chitosan/collagen hydrogels were prepared using glyoxal, a dialdehyde with relatively low toxicity as a crosslinking agent. Gels were freeze-dried and morphology was studied with varying concentration of both biopolymers. Cell studies revealed that glyoxal was cytocompatible at concentrations less than 1 mM for the time of exposure up to 15 h. Scanning electron micrographs showed that gels containing a high content of chitosan exhibited larger pores when compare with the gels containing lower chitosan concentrations. Crosslinked gels had even larger pores and a plate-like structure, while un-crosslinked gels showed a more uniform appearance [71].

## 5. Fabrication Methods

Fabrication methods for biobased porous materials more related to the choice of material. This can be classified into three main types. First, natural polymers, such as collagen and chitosan, are heat sensitive, so freeze-drying is mainly used to produce porosity, although electrospinning is also possible. Secondly, synthetic polymers, such as PLGA and PLA, often known as thermoplastics, so they can be fabricated by a wide variety of techniques. Bioceramics, such as hydroxyapatite tricalcium phosphate usually introduce as additives into polymeric matrices, since, pure ceramic matrices suffer from low hardness. Freeze-drying can be used to fabricate pure ceramic biomaterials, but this needs the use of sintering as a post-processing step which leads to additional porosity within the matrix walls [72].

Porous fabrication techniques can be categorized into two categories, designed manufacturing techniques and non-designed manufacturing techniques. Designed manufacturing technique includes 3D printing, rapid prototyping of solid free-form technologies. Non-designed manufacturing techniques include freeze-drying or emulsion freezing, solvent casting or particulate leaching, gas foaming, phase separation, electrospinning and combination of these techniques.

### 5.1. Solvent Casting and Particulate Leaching

**Figure 8:**
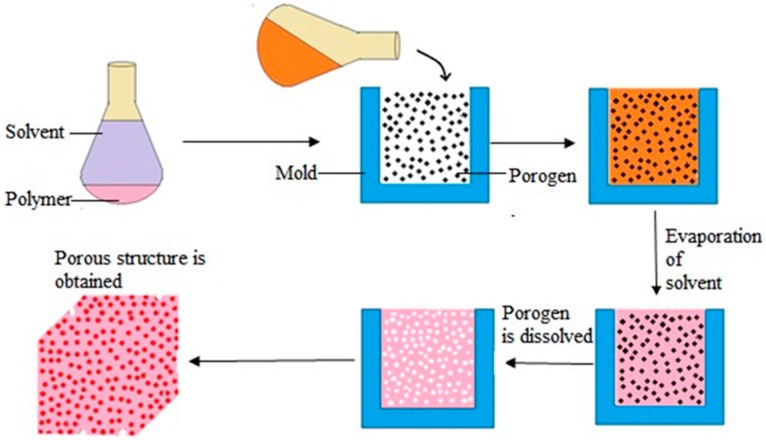
The schematic diagram of solvent casting and particulate leaching technique.

Solvent casting and particulate leaching is one of easy and cheapest way of porous scaffold fabrication. Figure 8 shows the detailed process of solvent casting-particulate leaching fabrication technique. The polymer is first dissolved in an organic solvent. Particles, mainly water soluble salts (e.g., sodium chloride, sodium citrate) with specific dimension are then added to the solution. After that, the mixture is poured into a mold of the desired shape. Next, the solvent is removed either by lyophilization or evaporation and allowed salt particles leached into the polymer matrix. Finally, the mold is dipped in a water bath for sufficient time to dissolve the salt particles, leached inside the polymer matrix. Porosity and pore size can be easily controlled by the amount and the size of salt particles added to the matrix. Though, difficulty confronted in the removal of leached salt particles from the matrix limits the thickness of the matrix to 0.5–2 mm [73].

Interconnected porous chitosan scaffold was prepared using sodium acetate particulate leaching method. Sodium acetate was mixed with chitosan solution and injected into the mold. Then the mold was freeze dried and lyophilized to evaporate the solvent. After that, washed with series of ethanol solution (100%, 90%, 80%, 70% and 50% *v*/*v*) sequentially for 2 h each and salt-leached in distilled water for 48 h. Finally, freeze-dried at −70 °C for 24 h and lyophilized for 24 h. It was observed that the porosity and pore interconnectivity increased with the ratio of sodium acetate. Moreover, with 90% sodium acetate ratio, many minute pores (7–30 µm) were formed between the main pores (200–500 µm) [74].

### 5.2. Thermally Induced Phase Separation

Thermally induced phase separation is a simple and versatile method for the preparation of microporous membranes. This method involves the dissolution of a polymer in a particular solvent having a high boiling point and low molecular weight at elevated temperature to form a homogeneous solution. Then the hot polymer solution is cast onto a mold followed by a cooling step. When a homogeneous solution at high temperature is cooled down, it induced solidification and phase separation into a polymer-rich phase and a polymer poor phase. After the solvent is removed by extraction or freeze-drying, a microporous structure is formed. This method is applicable for wide range of polymers, including those having poor solubility. The thermally induced phase separation process can be used to generate macro and microporous structure with an overall porosity as high as 90% [75,76].

Since this method has fewer influencing factors such as diluent, cooling rate, polymer concentration, and additives, it is easy for controlling membrane structures. As diluent is closely related to phase separation, different diluent cause different pore structures [77]. The advantages of this method are simplicity of the process, high reproducibility, low defects rate, high porosity and narrow pore size distribution [78].

Nano-hydroxyapatite/poly(l-lactic acid) composite scaffold was developed for bone tissue engineering and the morphologies, mechanical properties and protein adsorption capacities of the composite scaffolds was investigated. The porosity more than 90% was easily achieved and the pore sizes were able to adjust by varying the phase separation parameters [79].

PLLA/PLA scaffolds were prepared via thermally induced phase separation starting from ternary systems where dioxane as the solvent and water as the non-solvent. The porosity was within the range from 87% to 92%. Average pore size, pore distribution, pore interconnectivity and mechanical properties dependeded on the combination of the operating conditions such as solvent/non-solvent ratio, polymer concentration, remixing temperature and time [80].

### 5.3. Gas Foaming

**Figure 9:**
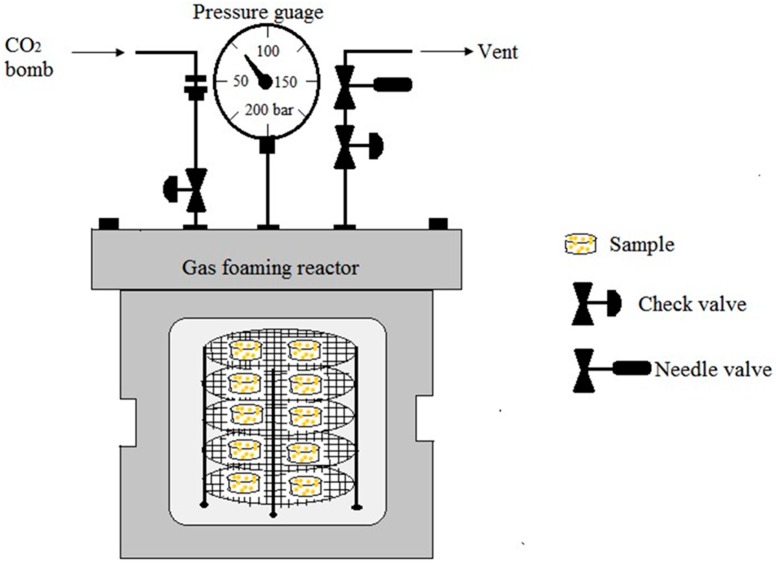
The schematic diagram of CO_2_ gas foaming device.

Gas foaming is being used to fabricate the polymers with high porosity without using any organic solvent [81]. This technique uses high-pressure CO_2_ for saturation of the polymer in an isolated chamber for a certain period of time. It needs high-pressure CO_2_ (800 psi) to saturate the polymer with gas [82]. When the polymer is saturated with CO_2_ at high-pressure, intermolecular interactions between CO_2_ and the polymer molecules become higher and causes a reduction of glass transition temperature of the polymer. Rapid depressurization causes thermodynamic instability and leads to form nucleated gas cells creating pores inside the polymer matrix. This technique is suitable for amorphous and semicrystalline polymers having relatively low *T*_g_ or *T*_m_ and high affinity for CO_2_ [83]. Instead of carbon dioxide, nitrogen gas can also be used for this method. The disadvantage is that it yields mostly a nonporous skin layer and closed pore structure [84]. This can be overcome by introducing a porogen such as salt particles (NaCl) to the polymer solution before gas foaming. Leaching of this salt particles, formed interconnected open pore structures in the polymer matrix. Porosity, pore interconnectivity can be controlled by altering the salt/polymer ratio and the particle size of the salt particles [85,86]. Figure 9 displays the schematic diagram of a CO_2_ gas foaming device.

The effect of high-pressure CO_2_ on the characteristics of elastin-based hybrid hydrogel was investigated. Compared to fabrication at atmospheric pressure condition, fabrication at high-pressure CO_2_ eliminated the skin-like layer formation on top of the hydrogel and formed larger pores with an average pore size of 78 ± 17 µm. However, the swelling ratio of the hydrogels fabricated at high-pressure CO_2_ decreased due to a higher degree of cross-linking. In addition, dense gas CO_2_ substantially increased the compressive and tensile modulus of fabricated hydrogels [87].

### 5.4. Emulsion Freeze-Drying

**Figure 10:**
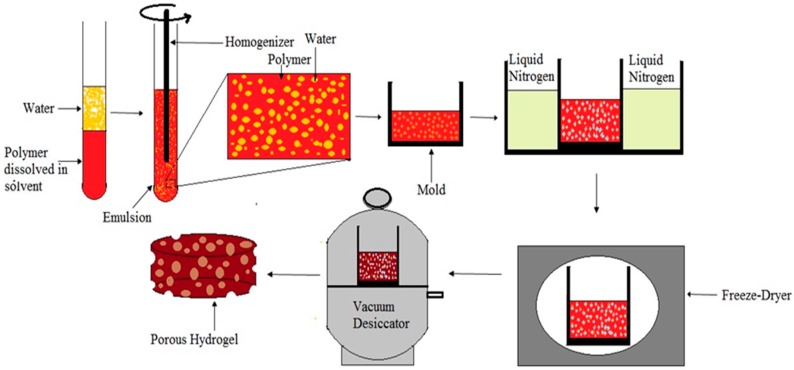
The schematic diagram of emulsion freeze-drying process.

In this method polymer is dissolved in its solvent and water is added. Then polymer solvent solution and water homogenized to form an emulsion. Before the separation of two phases, the emulsion is rapidly cooled to lock in the liquid state structure. Finally, solvent and water are removed by freeze-drying [88]. Figure 10 shows the schematic diagram of emulsion freez-drying process. This technique can be used to obtain porosity level above 90% and to control the pore size for targeted application. Porosity and pore structure can be controlled by polymer concentration, solvent, and water phase percentage, and freeze-drying parameters [89]. This technique is advantageous due to lack of leaching step but, the addition of organic solvent is a concern for the tissue engineering applications [90].

Hydroxyapatite/poly(hydroxybutyrate-*co*-valerate) composite scaffold was fabricated through the emulsion freezing/freeze-drying process and effect of polymer solution concentration, solvent and water phase on the morphology of composite scaffold was investigated. It was observed that at the same volume fraction of the water phase, the porosity of scaffolds decreased with increasing the polymer concentration. When the volume fraction of the water phase was increased, the porosity was found to be increased. It was reported that the produced scaffolds were highly porous with interconnected porous structures. Scaffolds exhibited pore sizes ranging from several microns to around 300 µm [91].

### 5.5. Rapid Prototyping Technique

**Figure 11:**
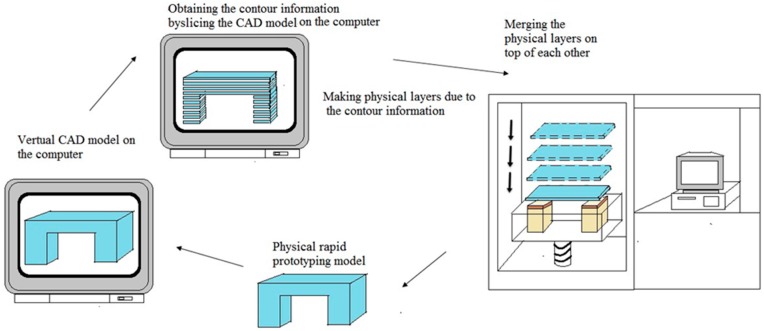
The schematic diagram of rapid prototyping technique.

Rapid prototyping, generally known as solid freeform fabrication technique and one of the most promising techniques for designing and producing scaffolds with 100% interconnected pores, fully computer controlled architecture with high porosities [92,93]. The inherent limitations such as long fabrication periods, incomplete removal of residual chemicals or volatile porogenic elements, labor intensive processes, poor repeatability, insufficient interconnectivity of pores and thin wall structures, irregularly shaped pores, of the conventional methods have led to use the rapid prototyping techniques to customize design and fabricate 3D porous scaffolds [94,95]. All current rapid prototyping techniques are based on the use of computer-aided design information that is converted to a stereo lithography type file format. Rapid prototype machine software processes this file to produce a solid model by a variety of processes. Starting from the bottom, the first layer of the physical model is created. The next layer is glued or bonded to the previous layer. This process is continued until the whole model is completed. Any supports are removed from the finished surface model and cleaned [96]. Figure 11 displays the schematic diagram of rapid prototyping technique. Main advantages of rapid prototyping process are rapid processing time, customization and efficiency [97]. Limitations of this techniques are high machine cost, high processing temperatures limiting the ability to process temperature-sensitive polymers, and need of multidisciplinary collaboration [98].

Hybrid poly(l-lactide)/chitosan scaffolds were developed using the rapid freeze prototyping technique. It was found that the mechanical properties of the scaffolds depend on the ratio of chitosan microspheres to poly(l-lactide) and cryogenic temperature used in the rapid freeze prototyping fabrication process. The results showed that scaffolds with greater porosity and enhanced pore size distribution compared to dispensing-based rapid prototyping technique [99].

### 5.6. 3D Printing

**Figure 12:**
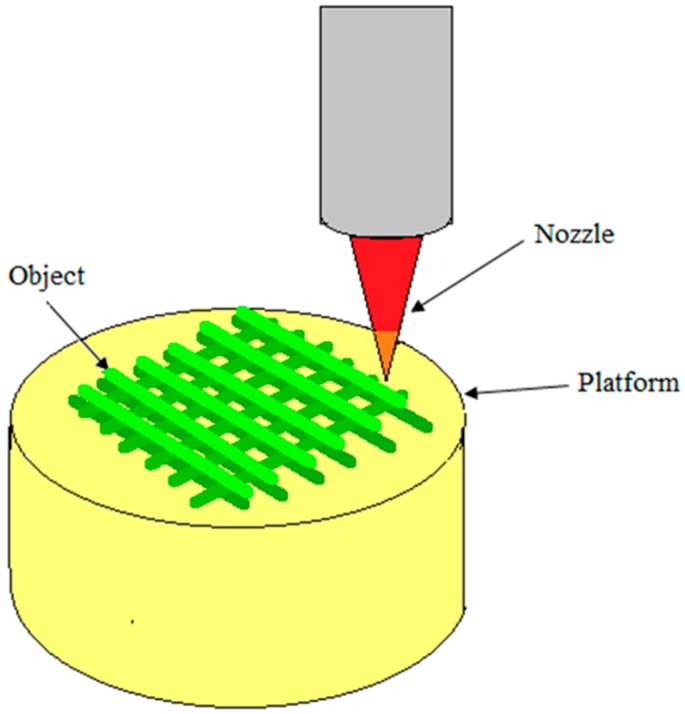
The schematic diagram of 3D printing technique.

3D printing, also known as additive manufacturing, and inkjet printing liquid binder is used to make a three-dimensional object from digital model data. This technique involves printing liquid binder to bind the loose powder to create 3D objects. Since biomaterials widely exist as solid or liquid form, most of them can be directly utilized in this technology. The first step of 3D printing is modeling of virtual model from computer-aided design (CAD) or animation modeling software. The machine uses these data as a guideline to print [100]. Then, a thin layer of powder is deposited onto the building platform. In the printing step, the machine reads the design from digital model data and a printer head selectively lay down liquid binder solution onto a powder bed to form the 2D pattern. This process is repeated layer by layer until the material/binder layering is completed and the final 3D model has been printed. Final objects are extracted from the powder bed by removing or dissolving the unbound powder [101]. Pore size and the spacing can be controlled by the pattern used. The advantage to 3D printing is the control of pore size and distribution. Both rapid prototyping (RP) and 3D printing technologies build models layer by layer using computer-aided design. But, there are still some differences such as 3D printers usually make smaller parts, 3D printing costs less, less material choices for 3D printers, 3D printers are less complex and easier to use than rapid prototyping machines. Figure 12 displays the schematic diagram of rapid prototyping technique.

Cylindrical scaffolds of five different designs were fabricated using a unique blend of starch-based polymer powders (cornstarch, dextran and gelatin) by 3D printing process. It was observed that the scaffold porosity corresponded to the designed porosities. A unique microporosity resulted due to the voids formed between granules or particles of the bulk material. Microporosity of the scaffolds of all designs were found to be within the range of 0.335–0.590. A highly interconnected porous network with suitable mechanical properties was fabricated using 3D printing process [102].

Indirect 3D printing protocol was employed to overcome the limitations of the direct technique for the preparation of porous scaffolds. 3D structures were fabricated by inkjet printing liquid binder droplets onto particulate matter. In indirect 3D printing protocol, molds are printed and the final materials are cast into the mold cavity. Scanning electron micrographs showed that well interconnected, highly open, uniform pore architecture (~100–150 μm), which is essential for uniform cell seeding, proliferation, growth, and migration in three dimensions [103].

### 5.7. Electrospinning Technique

**Figure 13:**
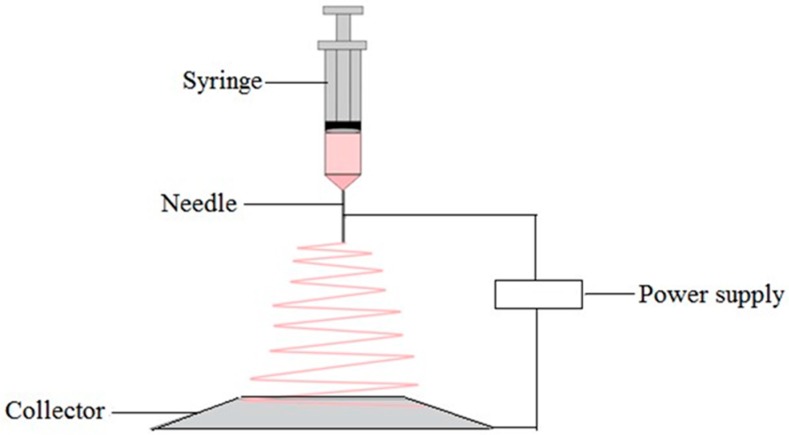
The schematic diagram of electrospinning technique.

A combination of two techniques namely electrospray and spinning is applied in electrospinning technique to form loosely connected 3D porous mats with high porosity and high surface area. A high electric field is applied to a fluid or melt which may extrude from a metallic syringe needle and acts as one of the electrodes as shown in Figure 13. When the electrostatic forces overcome the liquid surface tension forces the droplet comes to the end of the needle and deformed [104]. Then a fine, charged jet of the polymer solution is ejected from the tip of the needle to the counter electrode leading to the formation of continuous fibers. The fiber diameter and porosity of the matrix depend on the parameters such as voltage, polymer flow rate, the distance between the needle and the plate, and polymer concentration in the solution [105]. A wide range of polymers can be used in this technique such as synthetic polymers, natural polymers or a blend of both. The most important thing in electrospinning is that it can be used with various polymers, both in solution and in melt form. In melt electrospinning, it does not require the dissolution of the polymer in organic solvent, therefore it is environmentally safe and no mass loss due to solvent evaporation [106]. Electrospinning can be used to encapsulate drugs to the fibers. Also, this technique is the most economical way of producing nanofibers [107]. The disadvantage of this technique is limited control of pore size. The pore size of the matrix depends on the fiber diameter. Fibers with small diameter lead to form smaller average pore sizes. To overcome this limitation, a dual electrospinning setup has been developed with the additional stream of polymer which acts as a sacrificial fiber to increase the void space in the matrix [108,109].

A porous scaffold consisting of biopolymer nanofibers is one of the most promising candidates for tissue engineering applications. Immiscible biopolymers of gelatin and polycaprolactone were first electrospun to form composite fiber of gelatin/polycaprolactone. A leaching method was carried out to generate porous nanofibers to selectively remove the water soluble component of gelatin. After the leaching treatment grooves, ridges, and elliptical pores were appeared on the surface as well as inside of the resultant individual nanofibers [110].

Electrospun poly(ε-caprolactone)/chitosan nanofibers were prepared to study the effects of chitosan concentration on the bovine serum albumin (BSA) protein release behavior. Poly(ε-caprolactone) (PCL) and chitosan nanofibers with different ratios of chitosan were electrospun with using formic acid/acetic acid solvent system. Based on the scanning electron micrograph images, with increasing the chitosan content in nanofiber exhibited a higher fiber diameter and pore size. In addition, compared to PCL/chitosan nanofibers, PCL/chitosan/BSA nanofibers showed higher fiber diameter and larger pore size. Results showed that the chitosan ratio affected significantly in the protein release profile from the PCL/chitosan/BSA blend [111].

## 6. Conclusions

Each biopolymer molecule has a unique primary structure. Material-specific properties are due to the unique primary structure that they possess. In this review, we discussed two classes of biopolymers, namely, natural biopolymers (chitosan, cellulose, collagen) and synthetic biopolymers (PLA, PLGA) and their composite materials (chitosan/PLA, PLA/Cellulose, cellulose/chitosan, chitosan/PLGA, chitosan/collagen). Chitosan is a widely used biopolymer with attractive properties. It has been widely used to fabricate porous membranes, hydrogels, scaffolds and microparticles using a variety of simple fabrication techniques. Development of highly porous structure in cellulose is important because of their potential uses in tissue engineering, building insulating, antimicrobial food packaging applications, and, automotive, packaging and aircraft industries. Collagen can be easily fabricated into various forms such as sheets, sponges, tubes, fleeces, powders, injectable solutions, and dispersions. PLA has been widely using to fabricate porous scaffolds for tissue engineering applications. PLGA, a synthetic biopolymer which dissolved in a wide range of common solvents. Fabrication methods such as phase inversion, supercritical CO_2_, and thermally induced phase separation are widely used to fabricate porous PLGA scaffolds. Porosity, pore size and pore connectivity of fabricated biomaterials vary depending on the biomaterial, processing method and process conditions. All these factors can be controlled and varied depending on which application *is* needed. The properties of composite materials are superior to the properties of the individual components which it is constructed. Extreme modifications of the properties of biomaterials can be achieved by producing nanobiocomposites. Porous fabrication methods are two types; designed manufacturing techniques and non-designed manufacturing techniques. Natural polymers are heat sensitive and normally fabricated by methods with no heat generation. Synthetic biopolymers are often known as thermoplastics, so they can be fabricated by a wide range of techniques. Each fabrication method has its own advantageous and disadvantageous. A modern method for creating porous structures using biodegradable fibers by electrospinning is the latest development in this field. This method also has disadvantages like limitations of controlling the pore size. 3D printing, rapid prototyping, solvent casting and particulate leaching techniques are most suitable for controlling porosity and pore diameter as compared to other fabrication methods.

Biopolymer offers developers the tremendous flexibility to design porous matrices to broaden its applications in different areas day by day. In view of broadening the scope of biobased porous polymer applications, it is vital to take full advantageous of unique structure and properties of biopolymers to develop novel materials with pioneering new features.

## Figures and Tables

**Table 1:** An overview of pore characteristics of biomaterials fabricated with different methods.

Biopolymer	Fabrication Method	Application	Pore Characteristics	Reference
**Chitosan**	Freeze-drying	Scaffold	Polygonal pores formed with low molecular weight chitosan and elongated pores formed with high molecular weight chitosan. Average pore sizes of scaffold were approximately 60–90 μm.	[10]
**Chitosan**	Liquid hardening	Scaffold	Pore diameter decreased with increasing the stirring rate and decreasing the concentration of chitosan. Average pore sizes of 200–500 μm and 80% porosity could be obtained by varying the concentration of chitosan and the stirring rate.	[13]
**Chitosan**	Dense gas CO_2_	Scaffold	The porous structure obtained without formation of a nonporous skin layer. The average pore size in the scaffold produced at 60 bar and 4 °C was 30 to 40 μm using glutaraldehyde and genipin as crosslinker, respectively.	[15]
**Chitosan**	Supercritical CO_2_	Scaffold	Under optimum condition (CO_2_ pressure of 250 bar, 45 °C, 5 g/min CO_2_ flow rate for 2 h) that yielded 87.03% porosity. The pore sizes were in the range of 20–100 μm.	[16]
**Chitosan**	Liquid hardening	Scaffold	Pore sizes from 70 to 900 µm were obtained when transferring the stirred chitosan solution to sodium hydroxide solution. Macroporous chitosan scaffold with porosity 85% ± 2% was obtained.	[17]
**Cellulose**	Supercritical CO_2_	Polymeric foam	Pore size increased as decreasing the pressure and pore morphology varied with the depressurization rate.	[22]
**Cellulose**	Supercritical CO_2_	Antimicrobial food packaging	Mean pore size decreased with increasing the operative pressure and decreasing the operative temperature. Porosity increased with decreasing the pressure and with increasing the temperature.	[24]
**Cellulose**	Infrared laser	Scaffold	Pore size varied with adjusting the distance between specimen and laser focus. Patterned macropores with smooth surface and diameter larger than 100 μm was introduced to the scaffold with this method.	[25]
**Collagen**	Freeze-drying (using ice particulates as templates)	Scaffold	Two types of pores formed; one from the negative replica of ice templates and other from ice crystals developed by freeze-drying. Pore size decreased with decreasing the freezing temperature. The micropatterned pores of the scaffold can be controlled by designing a desirable micropattern for the ice template.	[30]
**Collagen**	Freeze-drying (mixing with ice particulates)	Drug delivery system	Interconnected pore structure obtained with pore size equivalent to ice particulates (150–250 µm). All the scaffolds had large controlled pore structure.	[31]
**Poly(l-lactic acid)**	Solvent-casting and particulate leaching	Scaffold	Two ranges of pore size formed using two particle sizes of NaCl as porogen: 150–250 µm and 251–425 µm. Pore structures were formed after Poly(l-lactic acid) dissolved in chloroform was dropped over the salt and leached with distilled water.	[35]
**PLA**	Phase-separation	Scaffold	Open porous PLA network formed with pore sizes greater than 100 µm and porosities of about 86%–94%.	[37]
**Poly(l-lactic acid)**	Solvent casting and particulate leaching	Scaffold	Interconnected pore structure formed with pre-designed pore sizes (280–450 µm) and porosity >94%.	[38]
**PLA**	CO_2_ blowing with the application of ultrasound	Scaffold	Interconnectivity of pores improved by ultrasound (by breaking the pore walls of closed pores). The diameters of the closed pores were from 30 to 70 µm. After the ultrasound treatment, pore sizes changed to 30–90 µm due to the formation of interconnected pores.	[39]
**PLA**	Solid state extrusion combined with porogen (NaCl) leaching method	Scaffold	Interconnected porous architecture formed with high connectivity exceeding 97% and with enhanced porosity over 60%. Smaller pore sizes (9 µm) were resulted due to the fragmentation of bulky NaCl during the processing.	[40]
**PLGA**	Phase inversion	Scaffold	Microporous interconnected architecture formed on the surface and within the bulk. The total porosities were 32.19% ± 11.4% and 72.24% ± 4.0% for the control (nonporous) and porous scaffolds, respectively.	[44]
**PLGA**	Supercritical CO_2_	Scaffold	Highly interconnected pores formed with relative pore densities ranging from 0.107 to 0.232 and porosities as high as 89%. The pore sizes were within the range from 30 to 100 μm.	[45]
**PLGA**	Thermally induced phase separation	Scaffold	Macropores with average diameter ~100 µm and interconnected micropores of 10–50 µm diameter formed with porosity > 93%. Tubular pores consited of radially oriented.	[46]
**PLGA**	Multi-emulsion method	Drug delivery system	Incorporation of pH-sensitive drug release activator increased the average pore diameter and surface area of microparticles in acidic medium. The average pore diameters of the microparticles at pH 7.4 were within the range of 11, 12, and 27 nm, respectively. It decreased at pH 6.0 to 13, 23, and 120 nm.	[47]

**Table 2:** An overview of pore characteristics of biocomposite materials fabricated with different methods.

Biocomposite Material	Fabrication Method	Application	Pore Characteristics	Reference
**Chitosan/PLA**	Melt molding and particulate (NaCl) leaching	Scaffold	The pore sizes were larger than 100 µm and all the pores including inner pores were interconnected. Porosity increased with the weight fraction of NaCl.	[52]
**Chitosan/PLA**	Freeze drying	Scaffold	Scaffold with interconnected porous structures and pore size around 100–500 µm was obtained. The pore size of the scaffolds decreased with increasing lactic acid/chitosan feed ratio. The chitosan scaffold had a porosity of 62.3% and pore size of 500 µm, and the lactic acid/chitosan scaffold (4:1, wt/wt) had a porosity of 34.37% and pore size of 100 µm.	[53]
**Chitosan/PLA**	Mold casting/infrared dehydration	Scaffold	Well-distributed 0.2 µm pores on the surface of the conduit was formed.	[54]
**PLA/nanocellulose**	Electrospinning	Release of nonionic compounds	There was no significant difference in the mean pore size between the nonwoven fabrics electrospun from PLA containing 0% and 1% cellulose nanocrystals. The mean pore size increased twice as big with PLA containing 10% cellulose nanocrystals. The mean pore sizes of the PLA nonwoven fabrics with 0%, 1% and 10% of cellulose nanocrystals were 0.48 ± 0.04 µm, 0.51 ± 0.08 µm and 0.94 ± 0.14 µm, respectively.	[59]
**Cellulose/chitosan**	Freeze drying	Sorption of trimethylamine and metal ions	The mean pore diameter was within the range of 100–300 μm. The pore diameters decreased with increasing chitosan concentration.	[60]
**Cellulose/chitosan**	Freeze drying	Dye adsorption	The beads were nanoporous with pore sizes from 10 nm to 20 nm.	[62]
**Bacterial cellulose nanofiber/chitosan**	Freeze drying	Scaffold	After the bacterial cellulose was treated by chitosan, porous structure remained but pore sizes became larger. Nanofibrous bacterial cellulose and bacterial cellulose/chitosan composite had well interconnected pore network structure.	[63]
**Chitosan/PLGA**	Electrospinning	Scaffold	In the electrospinning process, the spinning parameters, solution viscosity, polymer concentration, applied voltage, and flow rate highly influenced the porosity and pore size distribution of the composite material.	[64]
**Chitosan/PLGA nanocomposite**	Electrospinning and unidirectional freeze-drying	Scaffold	The porosity was found to be more than 96% and it decreased with increasing the chitosan concentration.	[65]
**Chitosan/PLGA nanocomposite**	Electrospinning and freeze drying	Scaffold	The porosity of chitosan/PLGA nanocomposite scaffolds decreased with increasing the chitosan solution concentration and electrospinning time.	[66]
**Chitosan/collagen**	Freeze drying	Scaffold	The chitosan scaffold showed the pore sizes between 500 and 700 µm while the chitosan/collagen composite scaffold showed a smaller pore sizes of 100–400 µm. The addition of collagen decreased the pore size of the composite scaffold. All samples composed of different proportions of chitosan and collagen showed porosities higher than 90%. The addition of collagen did not change the porosity.	[68]
**Chitosan/collagen**	Freeze drying	Scaffold	The mean pore size of the scaffold increased from 100 μm to >200 μm by crosslinking with glutaraldehyde. Elongated pores were formed with high concentration of glutaraldehyde. Refreeze-drying induced the fusion of some smaller pores to generate larger ones.	[69]
**Chitosan/collagen**	Freeze drying	Scaffold	At the highest chitosan/collagen ratio (75/25), the gels showed a sponge-like structure with larger pores than the gels containing lower chitosan content for both crosslinked and uncrosslinked scaffolds.	[71]
